# Impact of treatment with GLP‐1RAs on suicide attempts in adults persons with type 2 diabetes: A retrospective comparative effectiveness study based on a global TriNetX health research database

**DOI:** 10.1111/1753-0407.13547

**Published:** 2024-03-19

**Authors:** Mahmoud Nassar, Anoop Misra, Zachary Bloomgarden

**Affiliations:** ^1^ Department of Medicine, Division of Endocrinology, Diabetes and Metabolism, Jacobs School of Medicine and Biomedical Sciences University at Buffalo Buffalo New York USA; ^2^ Fortis‐C‐DOC Centre of Excellence for Diabetes, Metabolic Diseases and Endocrinology, Diabetes Foundation (India), and National Diabetes Obesity and Cholesterol Foundation (NDOC) New Delhi India; ^3^ Department of Medicine, Division of Endocrinology, Diabetes and Bone Disease Icahn School of Medicine at Mount Sinai New York New York USA

**Keywords:** comparative analysis, depression, diabetes mellitus, DPP‐4 inhibitors, GLP‐1RA, retrospective cohort study, suicide attempts

## Abstract

**Objective:**

To assess the association between glucagon‐like peptide‐1 receptor agonists (GLP‐1RA) treatment and the risk of suicide attempts in people with type 2 diabetes (T2D), with a focus on subgroups with and without a history of depression or suicide attempts.

**Methods:**

This retrospective cohort study utilized TriNetX, a federated network of real‐world data. Using the Global Collaborative Network data, we collected electronic medical records from 113 health care organizations with 135 million patient records with 8 million with T2D, 83% from the United States. The four cohorts were identified based on age, medication, diagnosis, and presence of depression or suicide attempts. Analytic methods included measures of association and number of Instances, with propensity score matching employed to mitigate potential confounders. The primary outcome was the incidence of suicide attempts among people with T2D with GLP‐1RA treatment in comparison with dipeptidyl peptidase‐4 inhibitor (DPP‐4i) treatment.

**Results:**

People with T2D treated with GLP‐1RA consistently exhibited a lower risk of suicide attempts compared to those treated with DPP‐4i. This was particularly significant in people with a history of depression or suicide attempts. The risk and odds ratios were significantly lower in the GLP‐1RA‐treated cohorts than in DPP‐4i across all analyses.

**Conclusion:**

As compared with DPP‐4i, our analysis shows a protective effect associated with GLP‐1RA treatment on the risk of suicide attempts among people with T2D. However, further research, particularly prospective and randomized studies, is necessary to confirm these observations and understand the underlying mechanisms.

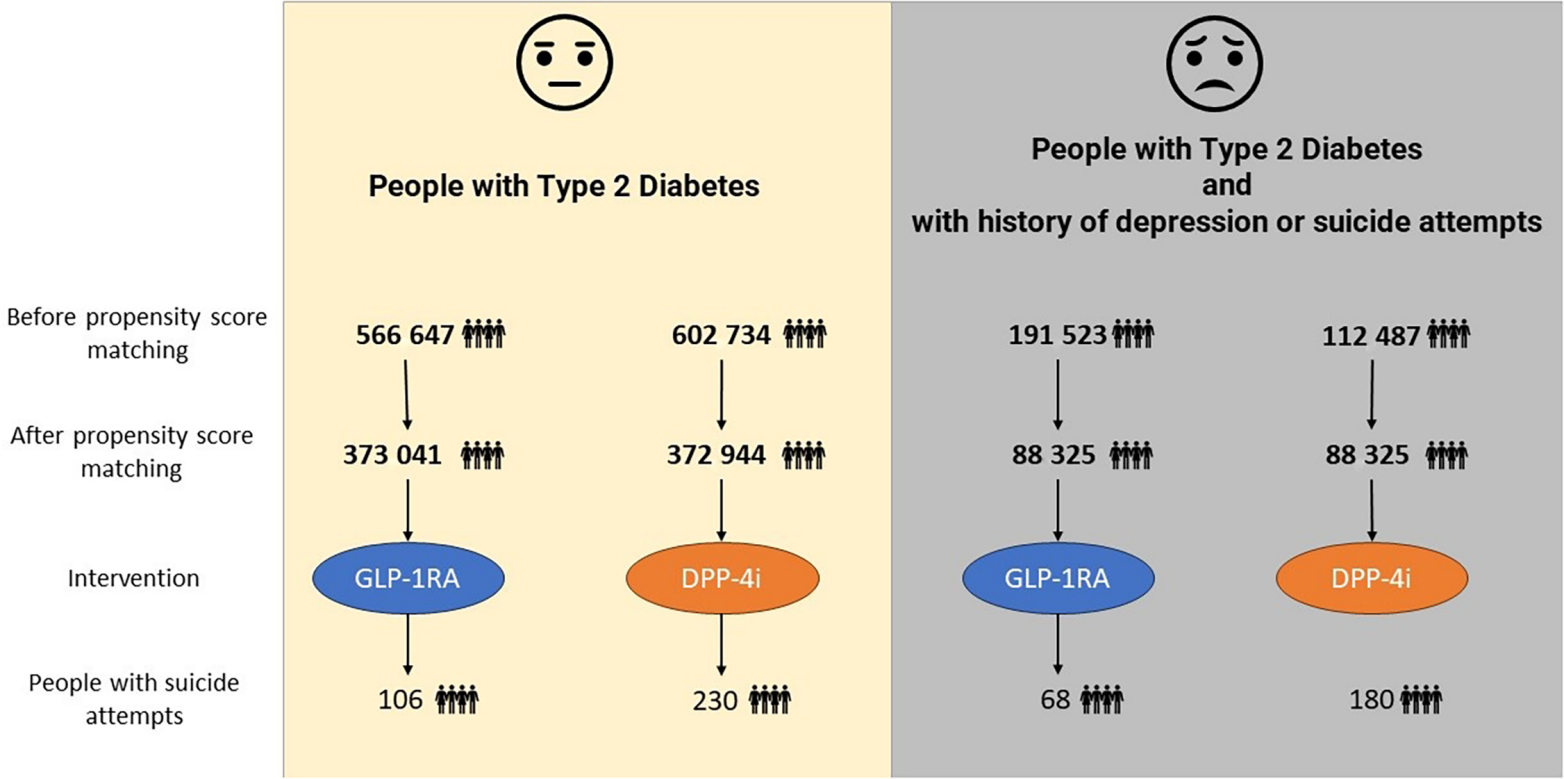

## Graphical abstract

Highlights

- This retrospective cohort study utilized the TriNetX global database and showed that the use of glucagon‐like peptide‐1 receptor agonists (GLP‐1RA) is associated with lower suicide risk in people with type 2 diabetes (T2D).
- Further, the use of GLP‐1RA was particularly associated with lower suicide risk compared with the use of dipeptidyl peptidase‐4 inhibitor (DPP‐4i) in people with T2D with a history of depression or suicide attempts.
- Overall, based on these data, GLP‐1RA might provide more protection for the risk of suicide attempts compared to DPP‐4i in people with T2D.

## 1 INTRODUCTION

The advent of glucagon‐like peptide‐1 receptor agonists (GLP‐1RA) has marked a significant advancement in the treatment of type 2 diabetes (T2D), offering not just glycemic control but also benefits in weight loss and cardiovascular risk reduction.^1^, ^2^, ^3^ However, the safety profile of these agents has come under scrutiny. Concerns have been heightened by reports of rare yet severe psychiatric adverse events, including suicide and self‐injury, potentially linked to GLP‐1RA treatments such as liraglutide and semaglutide, as indicated by warnings from health authorities like the Icelandic Medicines Agency.^4^, ^5^ An analysis of a subgroup within the Food and Drug Administration (FDA) Adverse Event Reporting System (FAERS) database showed a slight increase in the reporting odds ratio (ROR) for suicide and self‐injury in children who were taking GLP‐1RA (ROR: 2.50, 95% confidence interval [CI] 1.02–6.13, *p* = .05).^6^ Randomized controlled trials of semaglutide 2.4 mg weekly for obesity excluded persons with severe depression, but approximately 15% of persons enrolled in these studies were treated with antidepressants without evidence of a greater likelihood of adverse outcomes.^1^ Randomized controlled trials of liraglutide 3.0 mg daily for obesity similarly did not show an increase in the likelihood of depression or suicidality.^1^ The European Medicines Agency (EMA) is currently investigating the potential correlation between GLP‐1RA and the increased likelihood of experiencing suicidal thoughts and engaging in self‐harming behaviors.^5^ Also, the FDA is currently assessing the necessity of regulatory measures following the receipt of reports in its FDA FAERS regarding alopecia (hair loss), aspiration, and suicidal ideation among individuals using GLP‐1RA.^7^ It is noteworthy that concerns have been raised pertaining to the potential adverse psychiatric effects of bariatric surgery. Some studies have shown increased depression and suicide risk.^1^, ^2^, ^3^ Although other studies suggest the opposite may occur,^1^ with there being a potential for overall improvement in depression, a subset of persons show worsening, leading to suicidal behavior after these procedures.^2^ As yet, there is no firm conclusion on this issue. It is therefore important to determine whether real‐world evidence does or does not suggest that GLP‐1RA treatment of people with diabetes is associated with a greater risk of suicide.

## 2 METHODS

In this retrospective cohort study, data were sourced from the TriNetX Global Collaborative Network data, encompassing electronic medical records from 113 health care organizations with 135 million patient records. The database contains around 8 million individuals diagnosed with T2D, with over 83% residing in locations inside the United States. TriNetX is a global health research network that revolutionizes drug discovery and development by linking pharmaceutical companies, study sites, investigators, and people through a shared real‐world data platform (http://www.trinetx.com). It facilitates clinical and observational research by providing real‐time access to longitudinal clinical data and advanced analytics to enhance protocol design, site selection, and patient recruitment. Ensuring data privacy and compliance, TriNetX adheres to the Health Insurance Portability and Accountability Act and General Data Protection Regulation.

The overarching aim was to examine the impact of GLP‐1RA on suicide attempts among people diagnosed with T2D, specifically examining those with concurrent diagnoses of depression or suicide attempts to ascertain whether there was an adverse effect of GLP‐1RA.

This study exclusively involved individuals aged 18 and older diagnosed with T2D. Our analysis was segmented into two distinct parts, comprising four cohorts, differentiated based on the use of GLP‐1RA compared to dipeptidyl peptidase‐4 inhibitors (DPP‐4i) and the assessed risk level. DPP‐4i was selected as a comparable group because both are incretin‐based therapies. High‐risk individuals were identified as those with a history of suicide attempts, antidepressant medication use, or diagnoses of mood disorders such as dysthymic disorder or major depressive disorder, or those who had received treatments with selective serotonin reuptake inhibitors, nonselective monoamine reuptake inhibitors, tricyclic antidepressants, or monoamine oxidase inhibitors 5 years before the index event, which is starting either GLP‐1RA or DPP‐4i.

We conducted two comparative analyses across these four cohorts, utilizing specific inclusion criteria encompassing age, medication, and potential psychological factors. This study examines the incidence of suicide attempts within 5 years following the initiation of GLP‐1RA or DPP‐4i treatment.

1. Cohort 1 and 2: The first analysis evaluated the risk of suicide attempts in all people with T2D, comparing those treated with GLP‐1RA (Cohort 1, 566 645 people) against those treated with DPP‐4i (Cohort 2, 602 734 people).
2. Cohort 3 and 4: The second analysis targeted high‐risk people, with Cohort 3 comprising 191 523 people with T2D on GLP‐1RA treatment and Cohort 4 comprising 112 487 people with T2D on DPP‐4i treatment.

The analytical procedure involved two main steps: first, establishing the cohorts by selecting individuals who were at least 18 years old, had a history of T2D and were taking GLP‐1RA for GLP‐1RA cohorts and DPP‐4i for DPP‐4i for DPP‐4i cohorts second, excluding individuals with age <18 years and who were taking DPP‐4i treatment in GLP‐RA cohorts and GLP‐1RA treatment in DPP‐4i cohorts. In order to guarantee precision and pertinence during the analysis, it was necessary to establish explicit definitions for the index event, outcome criteria, and time frame.

The index event for each cohort, which serves as the starting point for the analysis, is defined by the initial date of GLP‐1RA or DPP‐4i treatment. Observations of outcomes began a day after the first occurrence of the index event and continued for 5 years after the index event. A total of 177 people from Cohort 1 and 274 people from Cohort 2 were excluded because they had a suicide attempt more than 5 years before the index event.

To gauge the relative frequency of suicide events with GLP‐1RA vs DPP‐4i, the risk difference per 100 000 persons and the ORs were calculated, both with 95% CIs. To mitigate potential confounders and biases, propensity score matching was employed based on patient characteristics like age, race, gender, depression‐related diagnosis, and use of antidepressant medications to create comparable groups for a balanced comparison. Outcomes were meticulously defined using a range of diagnostic and medication codes, focusing particularly on suicide attempts and the use of GLP‐1RA or DPP‐4i.

Ethical considerations were paramount, with all data handling and analyses conducted in compliance with relevant standards and regulations. Despite the study's retrospective nature and the use of deidentified data negating the need for specific patient consent, an appropriate ethics committee rigorously reviewed and approved the study's design and methods. This ensured adherence to ethical research principles and alignment with guidelines for secondary data analysis, reflecting the study's commitment to upholding ethical standards while contributing valuable insights into the treatment and management of T2D.

## 3 RESULTS

In the present study, we conducted a comprehensive comparative analysis of patient characteristics across different cohorts of people with T2D receiving GLP‐RA and DPP‐4i. The cohorts were divided based on their treatment regimens and depression risk characteristics.

Initially, Cohort 1 (T2D with GLP‐RA) and Cohort 2 (T2D with DPP‐4i) comprised 566 647 and 602 734 people, respectively. After propensity score matching, both cohorts were balanced with 373 218 people each, allowing for a more accurate comparison of demographic variables such as age, race, and gender. The same process was applied to Cohorts 3 and 4 (groups at high‐risk of suicide), which comprised 191 523 and 112 487 people, respectively. After propensity score matching, both cohorts were balanced with 88 325 people each.

Table 1 presents the results of the patient count and demographic characteristics before and after propensity score matching, showing that the procedure resulted in groups equivalent in age, sex, ethnicity, and depression‐related diagnoses. As compared with DPP‐4i, The GLP‐1RA cohort demonstrated fewer suicide attempts at baseline. As described under “Methods, “177 people were excluded from those taking GLP‐1RA, and 274 were excluded from those taking DPP‐4i because they had a suicide attempt more than 5 years before starting the medication. These differences were statistically significant (chi‐square = 20.9004, *p* = <.00001).

**TABLE 1:** Comparative analysis of characteristics of people with T2D before and after propensity score atching in GLP‐RA and DPP‐4i cohorts.

Cohort 1 and cohort 2 patient count before and after propensity score matching											
		Patient count before matching					Patient count after matching				
Cohort	1 ‐ T2D w GLP‐RA	566 647					373 218				
	2 ‐ T2D w DPP‐4i	602 734					373 218				
		Before Matching					After Matching				
Cohort	Demographics	Mean ± SD	People	% of cohort	*p* value	Std diff.	Mean ± SD	People	% of Cohort	*p*‐Value	Std diff.
1	Current Age	60.1 ± 13.1	566 645	100%	<.0001	0.8264	65.1 ± 11.1	373 218	100%	<0.0001	0.0198
2		70.8 ± 12.8	602 734	100%			64.8 ± 11.5	373 218	100%		
1	White		367 065	64.78%	<.0001	0.2172		226 858	60.78%	<0.0001	0.0112
2			326 554	54.18%				224 807	60.24%		
1	Female		347 005	61.24%	<.0001	0.1806		219 802	58.89%	<0.0001	0.0109
2			315 401	52.33%				217 799	58.36%		
1	Not Hispanic or Latino		305 547	53.92%	<.0001	0.1379		183 688	49.22%	<0.0001	0.0549
2			283 561	47.05%				173 455	46.48%		
1	Hispanic or Latino		106 559	18.81%	<.0001	0.1323		62 793	16.83%	0.0061	0.0063
2			83 911	13.92%				61 910	16.59%		
1	Black or African American		49 640	8.76%	<.0001	0.0189		32 696	8.76%	<0.0001	0.021
2			49 621	8.23%				34 945	9.36%		
1	Asian		15 168	2.68%	<.0001	0.1424		12 559	3.37%	0.0004	0.0082
2			33 068	5.49%				13 118	3.52%		
1	Mood [affective] disorders		116 267	20.52%	<.0001	0.2862		53 855	14.43%	<0.0001	0.0189
2			62 029	10.29%				51 399	13.77%		
1	Depressive episode		95 424	16.84%	<.0001	0.2539		44 168	11.83%	<0.0001	0.0171
2			51 035	8.47%				42 124	11.29%		
1	Major depressive disorder, recurrent		29 822	5.26%	<.0001	0.1996		10 427	2.79%	<0.0001	0.0177
2			9870	1.64%				9364	2.51%		
1	Bipolar disorder		13 418	2.37%	<.0001	0.0901		6530	1.75%	0.0655	0.0043
2			7109	1.18%				6323	1.69%		
1	Persistent mood [affective] disorders		8547	1.51%	<.0001	0.0527		4485	1.20%	0.0372	0.0048
2			5607	0.93%				4291	1.15%		
1	Unspecified mood [affective] disorder		8266	1.46%	<.0001	0.0865		3496	0.94%	<0.0001	0.0105
2			3548	0.59%				3130	0.84%		
1	Manic episode		474	0.08%	.227	0.0022		315	0.08%	0.2051	0.0029
2			466	0.08%				284	0.08%		

Cohort 3 and cohort 4 patient count before and after propensity score matching											
		Patient count before matching					Patient count after matching				
Cohort	5‐ T2D w GLP‐1RA (focus on high‐risk group)	191 523					88 325				
	6‐ T2D w DPP‐4i (focus on high‐risk group)	112 487					88 325				
		Before matching					After Matching				
	Demographics	Mean ± SD	People	% of cohort	*p* value	Std diff.	Mean ± SD	People	% of Cohort	*p*‐Value	Std diff.
3	Current Age	59.1 ± 12.8	191 523	100%	<.0001	0.829	65.7 ± 11.3	88 325	100%	0.0002	0.0175
4		69.8 ± 12.9	112 487	100%			65.9 ± 11.6	88 325	100%		
3	Not Hispanic or Latino		132 456	69.16%	<.0001	0.0965		58 467	66.20%	0.0543	0.0092
4			72 693	64.62%				58 849	66.63%		
3	White		124 916	65.22%	<.0001	0.0556		56 683	64.18%	0.0295	0.0104
4			70 366	62.56%				57 121	64.67%		
3	Female		122 639	64.03%	<.0001	0.1339		50 205	56.84%	< 0.0001	0.0386
4			64 692	57.51%				51 887	58.75%		
3	Black or African American		33 233	17.35%	<.0001	0.0784		13 725	15.54%	0.2682	0.0053
4			16 295	14.49%				13 894	15.73%		
3	Hispanic or Latino		15 015	7.84%	<.0001	0.0423		8295	9.39%	< 0.0001	0.0234
4			10 140	9.01%				7702	8.72%		
3	Asian		2694	1.41%	<.0001	0.1409		1854	2.10%	0.0013	0.0153
4			4053	3.60%				1665	1.89%		
3	Mood [affective] disorders		97 800	51.06%	<.0001	0.1885		39 005	44.16%	0.0035	0.0139
4			46 915	41.71%				39 615	44.85%		
3	Depressive episode		79 844	41.69%	<.0001	0.1584		31 862	36.07%	0.0115	0.012
4			38 281	34.03%				32 373	36.65%		
3	Major depressive disorder, recurrent		29 822	15.57%	<.0001	0.209		9010	10.20%	0.0337	0.0101
4			9870	8.77%				9282	10.51%		
3	Bipolar disorder		10 434	5.45%	<.0001	0.0546		4348	4.92%	0.4396	0.0037
4			4809	4.28%				4278	4.84%		
3	Persistent mood [affective] disorders		8487	4.43%	<.0001	0.0243		4081	4.62%	0.2743	0.0052
4			5562	4.95%				4178	4.73%		
3	Unspecified mood [affective] disorder		7308	3.82%	<.0001	0.0727		2408	2.73%	0.0277	0.0105
4			2858	2.54%				2561	2.90%		
3	Manic episode		373	0.20%	.0016	0.0117		171	0.19%	0.0792	0.0084
4			281	0.25%				205	0.23%		

Abbreviations: %, percentage; DPP‐4i, dipeptidyl peptidase‐4 inhibitors; GLP‐RA1, glucagon‐like peptide‐1 receptor agonists; N, Total number of people in a cohort; Std diff., standardized difference; T2D, type 2 diabetes.

### 3.1 Cohort 1 and 2 Analysis: Suicide Attempts in all People with T2D with GLP‐1RA vs those on DPP‐4i Treatment

In the first analysis, we assessed the risk of suicide attempts among people with T2D treated with GLP‐1RA and those treated with DPP‐4i. Cohort 1 (T2D with GLP‐1RA) consisted of 373 041 people, among whom 106 had a suicide attempt, resulting in a risk of 28.41 per 100 000. In comparison, Cohort 2 (T2D with DPP‐4i) included 372 944 people, with 230 experiencing a suicide attempt, for a risk of 61.67 per 100 000. The risk difference between the two cohorts was significant at −33 (95% CI: −43, −24) per 100 000 with an OR for those with GLP‐1RA vs DPP‐4i treatment of 0.461 (95% CI: 0.366, 0.58), *p* < .001 (Table 2).

**TABLE 2:** Comparative analysis of suicide attempt risks in people with T2D with GLP‐1RA treatment vs DDP‐4i.

Comparison	Cohort	Sample size (n)	People with outcome	Risk per 100 000	Risk difference (95% CI) per 100 000	*p* value	Odds ratio (95% CI)
T2D with GLP1‐RA vs T2D with DPP‐4i	1: T2D w GLP‐1RA	373 041	106	28.41	−33 (−43, −24)	<.001	0.461 (0.366, 0.58)
	2: T2D with DPP‐4i	372 944	230	61.67			
T2D with GLP1‐RA vs T2D with DPP‐4i (including only people with a history of depression or suicide attempts)	3: T2D with GLP‐1RA	88 325	68	76.98	−127 (−162, −92)	<.001	0.377 (0.285, 0.499)
	4: T2D with DPP‐4i	88 325	180	203.79			

Abbreviations: CI, confidence interval; df, degrees of freedom; DPP‐4i, dipeptidyl peptidase‐4 inhibitors; GLP‐1RA, glucagon‐like peptide‐1 receptor agonists; n, sample size; T2D, type 2 diabetes.

### 3.2 Cohort 3 and 4 Analysis: Suicide Attempts in People with T2D with GLP‐1RA Treatment vs DPP‐4i (including only people with high‐risk depression/suicide risk)

This analysis focused on people identified as high‐risk risk (with a history of depression or suicide attempts). Cohort 3 (T2D with GLP‐1RA) included 88 325 people, with 68 suicide attempts, equating to a risk of 76.98 per 100 000, and Cohort 4 (T2D with DPP‐4i) consisted of 88 623 people, with 176 suicide attempts, presenting a higher risk of 203.79 per 100 000. The risk difference was −127 (95% CI: −162, −92) per 100 000 with a *p* value of <.001. The OR for those with GLP‐1RA vs DPP‐4i treatment was 0.377 (95% CI: 0.285, 0.499) (Table 2).

## 4 DISCUSSION

In this population‐based study involving a large number of participants with T2D, those treated with GLP‐1RA exhibited a consistently lower risk of suicide attempts compared to those treated with a DPP‐4i. The high‐risk group of individuals with a history of depression or suicide attempts showed the same outcome. The risk ratios and ORs were significantly lower in the GLP‐1RA treated cohorts across all analyses, strengthening the argument that there is no adverse effect of GLP‐1RA treatment in increasing suicide attempts among the diabetic population.

The relationship between GLP‐1RAs and mental health is complex. Studies have reported a range of experiences from people using GLP‐1RAs, including both positive effects on mood and negative experiences such as heightened anxiety and difficulty sleeping.^8^ Real‐world pharmacovigilance analysis has not shown a disproportionate increase in suicide/self‐injury events overall associated with GLP‐1RAs, but nuances exist, with a reported increase in children (OR: 2.50, 95%CI 1.02–6.13, *p* = .05).^6^ Moreover, *post hoc* pooled data analysis from randomized controlled trials of liraglutide, a GLP‐1RA, showed similarly low rates of depression and anxiety between the liraglutide and placebo groups but did reveal a small numerical imbalance in suicidal ideation among liraglutide users.^4^ Based on information from the Icelandic Medicines Agency, the EMA is conducting a thorough examination that will study approximately 150 cases.^5^ There is some evidence that GLP‐1RA can penetrate the blood–brain barrier,^9^ and GLP‐1 receptors are present in the central nervous system,^10^ which may affect hunger signals and elevate neuropsychiatric risks^5^ or which, alternatively, may have psychiatric as well as metabolic benefits. After the submission of the present manuscript, Wang and coworkers published a similar analysis using the TriNetX database, showing that, compared with a propensity score matched group of 27,276 persons with diabetes taking all other diabetes medications, among the same number of persons taking GLP‐1RA for diabetes the likelihood of suicidal ideation was >60% lower, with similar findings among persons with obesity not having diabetes taking vs. not taking GLP‐1RA. The present study made the more specific comparison of GLP‐1RA with DPP‐4i, and compared the general population of persons with diabetes as well as those with prior history of depression or suicide attempts.^11^

The study's strengths lie in its large sample size and the use of a comprehensive global federated health research network, providing a robust dataset for examining the effects of GLP‐1RA on suicide attempts in diabetic people. Its detailed stratification of people into specific cohorts based on their medical history allowed for a nuanced analysis of the treatment's impact across different subgroups, enhancing the relevance and specificity of the findings. Additionally, using propensity score matching to control for confounders adds to the credibility of the results.

However, this study has several limitations. Its retrospective nature inherently restricts the ability to establish causality and residual confounding factors may not be accounted for or measured. People with T2D initiating GLP‐1RA may experience disparate care access compared to DPP‐4i users. Our findings indicate a statistically significant higher incidence of suicide attempts in patients subsequently prescribed DPP‐4i compared to GLP‐1RA, suggesting potential selection bias despite propensity matching. The reliance on electronic medical records and diagnostic codes may lead to misclassification or underreporting of outcomes like suicide attempts. Access to GLP‐1RA in the United States often has a high‐cost barrier, whereas the DPP‐4i have become much less expensive, a potential cause of selection bias. The study's setting within a specific network of health care organizations might limit the generalizability of the findings to broader populations. Furthermore, although the data were adjusted for several known confounders, unknown or unmeasured variables could still influence the results. The study did not consider other factors that might be related to suicide, such as blood glucose control levels and episodes of hypoglycemia, family history of suicide, and socioeconomic status. Lastly, the observational design cannot match the rigor of randomized controlled trials in determining treatment efficacy and safety, making it crucial to approach the conclusions to understand these contextual limitations.

## 5 CONCLUSION

This retrospective cohort study showed no evidence of an adverse effect of GLP‐1RA in increasing the risk of suicide attempts among people with T2D, especially those with a history of depression or suicide attempts. Analysis across various patient subgroups consistently showed that those treated with GLP‐1RA had significantly lower risk and fewer instances of suicide attempts compared to those treated with DPP‐4i. This study argues strongly against there being a potential for GLP‐1RA to cause an increase in suicide risk. These findings require further investigation to more fully understand the underlying mechanisms and broader clinical implications.

## FUNDING INFORMATION

This research did not receive any specific grant from funding agencies in the public, commercial, or not‐for‐profit sectors.

## DISCLOSURE

Anoop Misra is the associate editor and Zachary Bloomgarden is editor‐in‐chief of the journal and also coauthors of this article. They were excluded from the peer‐review process and all editorial decisions related to the acceptance and publication of this article. Peer‐review was handled independently by another editor‐in‐chief and the assigned associate editor.

## PATIENT CONSENT STATEMENT

Patient consent was not applicable to this study as it did not involve human participants.

## References

[1] Baggio LL , Drucker DJ . Glucagon‐like peptide‐1 receptor co‐agonists for treating metabolic disease. Mol Metab. 2021;46:101090. 32987188 10.1016/j.molmet.2020.101090 PMC8085566

[2] Jepsen MM , Christensen MB . Emerging glucagon‐like peptide 1 receptor agonists for the treatment of obesity. Expert Opin Emerg Drugs. 2021;26(3):231‐243. 34176426 10.1080/14728214.2021.1947240

[3] Taha MB , Yahya T , Satish P , et al. Glucagon‐like peptide 1 receptor agonists: a medication for obesity management. Curr Atheroscler Rep. 2022;24(8):643‐654. 35624390 10.1007/s11883-022-01041-7

[4] O'Neil PM , Aroda VR , Astrup A , et al. Neuropsychiatric safety with liraglutide 3.0 mg for weight management: results from randomized controlled phase 2 and 3a trials. Diabetes Obes Metab. 2017;19(11):1529‐1536. 28386912 10.1111/dom.12963 PMC5655710

[5] Cohen D . GLP‐1 receptor agonists: European drug regulator asks makers for evidence of self‐harm. BMJ. 2023;383:2906. 38084503 10.1136/bmj.p2906

[6] Chen C , Zhou R , Fu F , Xiao J . Postmarket safety profile of suicide/self‐injury for GLP‐1 receptor agonist: a real‐world pharmacovigilance analysis. Eur Psychiatry. 2023;66(1):e99. 38031404 10.1192/j.eurpsy.2023.2474 PMC10755578

[7] FDA . July–September 2023 | Potential Signals of Serious Risks/New Safety Information Identified by the FDA Adverse Event Reporting System (FAERS) . 2023 Available from: https://www.fda.gov/drugs/questions‐and‐answers‐fdas‐adverse‐event‐reporting‐system‐faers/july‐september‐2023‐potential‐signals‐serious‐risksnew‐safety‐information‐identified‐fda‐adverse

[8] Arillotta D , Floresta G , Guirguis A , et al. GLP‐1 receptor agonists and related mental health issues; insights from a range of social media platforms using a mixed‐methods approach. Brain Sci. 2023;13(11):1503. 10.3390/brainsci13111503 PMC10669484 38002464

[9] McClean PL , Parthsarathy V , Faivre E , Hölscher C . The diabetes drug liraglutide prevents degenerative processes in a mouse model of Alzheimer's disease. J Neurosci. 2011;31(17):6587‐6594. 21525299 10.1523/JNEUROSCI.0529-11.2011 PMC6622662

[10] Wong CK , McLean BA , Baggio LL , et al. Central glucagon‐like peptide 1 receptor activation inhibits toll‐like receptor agonist‐induced inflammation. Cell Metab. 2023;36:130‐143.e5. 38113888 10.1016/j.cmet.2023.11.009

[11] Wang W, Volkow ND, Berger NA, Davis PB, Kaelber DC, Xu R. Association of semaglutide with risk of suicidal ideation in a real‐world cohort. *Nat Med*. 2024;30(1):168–176. doi:10.1038/s41591-023-02672-2 PMC11034947 38182782

